# Psychographic segmentation to identify higher-risk teen peer crowds for health communications: Validation of Virginia's Mindset Lens Survey

**DOI:** 10.3389/fpubh.2022.871864

**Published:** 2022-07-22

**Authors:** Carolyn A. Stalgaitis, Jeffrey W. Jordan, Mayo Djakaria, Daniel J. Saggese, Hannah Robbins Bruce

**Affiliations:** ^1^Rescue Agency, San Diego, CA, United States; ^2^Virgina Foundation for Healthy Youth, Richmond, VA, United States

**Keywords:** peer crowd, adolescent, segmentation, health communication, equity, validation, psychographic

## Abstract

Audience segmentation is necessary in health communications to ensure equitable resource distribution. Peer crowds, which are macro-level teen subcultures, are effective psychographic segments for health communications because each crowd has unique mindsets, values, norms, and health behavior profiles. These mindsets affect behaviors, and can be used to develop targeted health communication campaigns to reach those in greatest need. Though peer crowd research is plentiful, no existing peer crowd measurement tool has been formally validated. As such, we developed and validated Virginia's Mindset Lens Survey (V-MLS), a mindset-based teen peer crowd segmentation survey to support health communication efforts. Using an online convenience sample of teens (*N* = 1,113), we assessed convergent and discriminant validity by comparing the V-MLS against an existing, widely-used peer crowd survey (I-Base Survey^®^) utilizing a multi-trait multi-method matrix. We also examined the V-MLS's predictive ability through a series of regressions using peer crowd scores to predict behaviors, experiences, and traits relevant to health communication campaign planning. The V-MLS demonstrated reliability and convergent and discriminant validity. Additionally, the V-MLS effectively distinguished teen peer crowds with unique health behaviors, experiences, and personal traits. When combined with appropriate information processing and campaign development frameworks, this new tool can complement existing instruments to inform message framing, tone, and style for campaigns that target at-risk teens to increase campaign equity and reach.

## Introduction

Health inequities are persistent. One challenge of equitably administering public health services lies in identifying population segments who are at elevated risk, and understanding how to reach them effectively with resources that meet their needs. Audience segmentation, the process of identifying homogeneous subgroups within heterogeneous populations, is necessary in public health communications to increase equity by allowing campaign planners to create content that is tailored to a higher-risk audience's needs and delivered *via* targeted media channels to reach them effectively (1). Though public health has slowly incorporated segmentation into campaign development and delivery, the field too often segments based on demographics such as gender, race, or ethnicity alone, which is insufficient and can lead to stereotyping, exclusion, and the reinforcement of disparities (2–4).

Recently, public health and social marketers have embraced the use of psychographics such as mindsets, values, and worldviews to segment audiences. Promising results indicate that using psychographics alone or in combination with demographics leads to more clearly-defined audience segments than demographics alone (2–7). In particular, psychographic segmentation identifies audiences with unique behavioral needs who can be reached *via* specific media channels (1–3, 6, 7). Additionally, psychographic segmentation can inform campaign development in a way that demographic segmentation alone cannot, as the worldviews, values, and mindsets that define psychographic segments can be incorporated into message framing and content to increase persuasion (8).

Psychographic audience segmentation is effective because it creates groups based on shared characteristics that directly influence behavior, rather than demographic characteristics that may only proxy direct behavioral influences (5, 8). Though a range of approaches can be used in psychographic segmentation, many cutting-edge public health communication campaigns have segmented teens and young adults using peer crowds. Peer crowds are macro-level, reputational subcultures with shared values, interests, media preferences, visual cues, and behavioral norms (9, 10). Broader than the peer groups with which a young person interacts on a daily basis, peer crowds represent shared culture and identity across communities (9, 11). Peer crowds are ideal for health communication audience segmentation because campaign content can address the specific behavioral norms of a targeted peer crowd, using the crowd's shared values and mindsets to increase message relevance and tailoring visual cues to increase appeal and information processing. Peer crowds also tap into a key factor in teen and young adult behavior—social influence (12)—by identifying groups of youth with shared norms who can be reached together and whose behavior influences each other. Finally, peer crowd campaigns can be delivered efficiently by focusing resources on media channels where crowd members naturally gather, increasing campaign exposure among the target crowd without increasing campaign waste (13, 14).

Peer crowds fulfill a symbolic role within adolescent culture, each representing a particular set of values and lifestyles that a youth can embrace or reject as they explore their place within the social environment (15–17). Crowds form through both selection (individuals with shared values, interests, and experiences seeking out each other) and socialization (crowd members reinforcing shared culture, mindsets, and behaviors as a means of maintaining identity and belonging) (18, 19). Peer crowds influence behavior by providing a prototype of normative crowd behavior, which a young person may emulate to signal to others their identity as a member of that crowd (20–22).

One way of understanding the role of values and mindset in adolescent peer crowd culture is through the mindsponge model (23, 24). Though initially developed to explain processes of acculturation and cross-cultural business (23), the mindsponge model has been applied to describe other psychosocial phenomenon (24) and can be a useful way to think about how a person's values and mindsets evolve during adolescence. As peers overtake the family as a teen's primary source of socialization and culture (25–27), the values and mindsets that the youth learned from their family may be challenged by new values held by their peers. The relevance, compatibility, and costs/benefits of these new values will influence if and how a teen incorporates these values into their comfort zone and eventually their mindset, as well as which previously-held values they may drop (23, 24). The values that pass a teen's filters will become part of their core beliefs, affecting behavior and which values they may accept in the future (24). This dynamic process may explain how youth develop peer crowd identifications *via* adoption of a crowd's cultural values, how a teen's peer crowd identification may evolve over time, and why peer crowds tend to fade in relevance in adulthood as other cultural contexts with their own value sets such as the workplace and family become a person's primary sources of socialization.

Peer crowds have been the subject of cross-disciplinary research for decades (Table 1). Research in the U.S. and in other, primarily Western, countries consistently identifies similar teen peer crowds with particular values, mindsets, interests, and behaviors across settings and methods. The Hip Hop and Alternative peer crowds reliably demonstrate elevated risk across a range of health topics including tobacco use, substance use, and mental health. Other crowds such as Country and Popular tend to have moderate risk levels, while Mainstream is typically at low risk across health topics.

**Table 1:** Common teen peer crowds (11, 18, 20, 28–54).

**Crowd Name**	**Description**	**Risk**
**Hip Hop** *(other names from previous literature: Urban, Gangsters, Rappers)*	- Driven to succeed despite perceived obstacles or struggles; value confidence, authenticity, strength, and family - Often dress in a style popularized by rappers, including snapback or beanie hats and stylish sneakers - Typically prefer hip hop, rap, and R&B music genres	- Greater risk for cigarettes, cigarillos, vapes, hookah, alcohol, marijuana, other drugs; depression and anxiety - Report higher levels of childhood adversity
**Alternative** *(other names from previous literature: Rebels, Skaters, Goth, Emo, Hipsters, Rockers, Metal Heads, Punks)*	- Rebel against authority; value uniqueness and self-expression, often displayed *via* extreme personal style choices - Participate in the arts and support progressive social causes - Typically prefer rock, alternative, metal, punk, or indie music genres	- Greater risk for cigarettes, alcohol, marijuana, other drugs; weight-control behaviors, physical inactivity, obesity; self-harm, suicidal ideation and attempts, low self-esteem, depression, anxiety, and loneliness - Report higher levels of childhood adversity
**Country** *(other names from previous literature: Farmers, Rural, Cowboys, Rednecks, Hicks)*	- Often from rural areas; enjoy outdoor activities (e.g., hunting, fishing) and may be involved in farming or ranching - Value community, patriotism, and individual liberty - Respect hard work and hands-on or manual labor	- Greater risk for smokeless tobacco, cigarettes; poor nutrition, obesity, and physical inactivity - Lower risk for alcohol, marijuana, other drugs; suicidal ideation and attempts, and depression - Report lower levels of childhood adversity
**Popular** *(other names from previous literature: Elites, Jocks, Partiers, Preps, Social, Cheerleaders)*	- Outgoing, social, high-status youth often involved in school activities, particularly athletics; value living in the moment and novel experiences - May be academically successful, but prioritize social status over academics - Prefer latest fashion trends and Top 40 popular music	- Greater risk for vapes, alcohol, and indoor tanning - Lower risk for cigarettes; obesity, physical inactivity, poor management of diabetes; depression, anxiety, low self-esteem, loneliness, and suicidal ideation and attempts - Report lower levels of childhood adversity
**Mainstream** *(other names from previous literature: Brains, Normals, Regulars, Quiet, Academic, Homebody)*	- Academically-oriented; value following rules, stability, and helping others - Positive relationships with authorities and adults; less socially popular with peers - Typically unremarkable style and music preferences	- Lower risk for tobacco, alcohol, marijuana, other drugs; poor nutrition; depression, anxiety, low self-esteem, loneliness, suicide attempts; and indoor tanning

Peer crowds provide a purposeful means of audience segmentation that maximizes equity by allowing communication campaigns to create tailored content delivered *via* targeted channels to higher-risk crowds (13). Experimental studies indicate that peer crowd-matched educational materials can influence behavioral attitudes and beliefs (55–57). In real-world applications (see Supplementary Figure 1 for examples), youth and young adult peer crowd-targeted campaigns have been associated with high levels of awareness, positive receptivity, and desired behavior change (14, 58–63). Peer crowd segmentation for public health communication represents a promising means of increasing equity by focusing resources on crowds with disproportionate risk. However, this approach requires both an effective and practical means of distinguishing members of a given crowd, and an understanding of the values, interests, visual cues, and preferred media channels of the crowd to inform message content, framing, style, and placement.

Despite convergence across studies, no “gold standard” exists for measuring peer crowd identity (64). Existing methods each have benefits and drawbacks for audience segmentation purposes. Ethnographic methods, wherein researchers observe and interview teens in a single community to understand the prevalent crowds, provide detailed portraits of peer crowds but are not sufficiently generalizable or scalable to be used in campaign audience segmentation (50, 65–68). Other studies use a peer-nominated social-type rating approach, whereby students at a given school place their classmates into peer crowds (16, 20, 43). This approach also lacks scalability and generalizability as it requires students to know each other sufficiently well to assign crowd affiliation, and agreement between raters is often low (43, 64). Furthermore, peer ratings focus on others' perceptions of an individual's crowd rather than the individual's self-perception, which is of greater relevance to public health communication segmentation as it more directly influences behavior (31) and avoids stereotyping.

Other approaches directly measure an individual's identity by asking youth to select their peer crowd affiliation from a list of names. Iterations of this approach include forcing selection of a single crowd, allowing selection of multiple crowds, or indicating degree of affiliation with multiple crowds, and may or may not include detailed crowd descriptions (28–32, 34, 42–45, 47, 52, 54–57, 69–71). For example, the Peer Crowd Questionnaire and College Peer Crowd Questionnaire ask participants to confirm if each crowd from an established list exists in their area (e.g., Jocks, Brains, Burnouts, Populars, Non-Conformists, or None/Average), and with which of the crowds they identify (18, 33, 35, 38, 72–75). Though more scalable for audience segmentation, using crowd names suffers from desirability and other biases related to the names and descriptions used. Colloquial crowd names often differ across communities, and individuals may avoid selecting crowd names they perceive as undesirable or having negative connotations even if they personally identify with the group that the name was intended to represent (15, 31, 43, 64). Additionally, forcing youth to select a single crowd fails to account for the multifaceted nature of identity, as many individuals identify with more than one crowd (31, 32, 34, 37, 45). Finally, name-based self-reports provide none of the insight needed to create a targeted campaign such as a crowd's values, worldviews, mindsets, or visual cues, leaving campaign planners without the insights necessary to tailor content.

To address shortcomings of direct measures, indirect measures ask youth to report characteristics associated with peer crowds and assign affiliation using these data. Several studies have collected data on activities (e.g., studying, athletics, partying) or adjective pairs (e.g., quiet/loud, popular/unpopular, athletic/not athletic) and applied cluster analysis techniques to assign youth to peer crowd clusters (76–80). Though a cluster analysis approach creates homogeneous segments, it is unclear if the clusters reflect interactional crowds with shared behavioral norms, making application to health communication campaigns limited. Other indirect approaches tap into the visual cues of peer crowd identity (11, 53). One example, the I-Base Survey^®^, includes a grid of photos of unknown youth with each photo representing a single peer crowd based on qualitative research (36, 81). Respondents select photos that would and would not fit with their group of friends, resulting in a score indicating strength of identification with each crowd (11, 13, 37, 40, 41, 58–63, 82). While formal validation studies of the I-Base Survey have not been published, the instrument has been used extensively across U.S. communities and over time with consistent results, indicating the I-Base Survey has a level of applied validity and utility in audience segmentation (11, 13, 36, 37, 40, 41, 82–85). This approach overcomes issues associated with asking individuals directly and forcing identification with a single crowd, and provides insight into the visual cues that should be applied to a communication campaign to attract the attention of the targeted crowd. However, photos must be updated regularly to stay abreast of fashion trends and must be adjusted to reflect the racial and ethnic demographics of the community.

While several approaches exist to measure peer crowd identification, to date there is no formally validated, scalable, and replicable peer crowd segmentation tool. With this in mind, we developed and validated a mindset-based teen peer crowd measurement tool that addresses many of the shortcomings of existing instruments, and more directly measures the psychographics that make peer crowds unique segments that can be effectively targeted in health communications. We focused on values and mindsets as these characteristics influence behavior, are predictive of health risk, and can effectively segment audiences (23, 24, 86–89). To develop the instrument, we conducted formative research leading to the large-scale validation survey described in detail here. The result is a survey that effectively segments teens into distinct psychographic subgroups with unique health risk profiles, while also providing insight into the values and mindsets that should be incorporated into a health communication campaign targeting any given crowd. As the study described herein was conducted in Virginia, we refer to the instrument as Virginia's Mindset Lens Survey (V-MLS). However, as peer crowds transcend geographic boundaries, we believe V-MLS findings are likely generalizable across the U.S. and anticipate future work to establish the survey's applicability outside the state. Additionally, future cross-cultural adaptation work could ensure applicability of the instrument in other countries, where cultural differences require care to ensure local crowds and values are reflected accurately. The V-MLS represents an evolution in peer crowd measurement methods that can complement existing strategies and tools to identify, target, and tailor efforts to higher-risk segments to address health disparities.

## Materials and methods

### Study approach and design

Prior to data collection, we identified key parameters for instrument creation. First, we sought to measure identification with the peer crowds commonly identified in existing literature across different geographies, times, and methodologies (Table 1). We selected the I-Base Survey as our comparison instrument, given the widespread use of this survey both to understand peer crowd risk (11, 13, 36, 37, 40, 41, 82) and to create and evaluate peer crowd-targeted communication campaigns (58–63). Therefore, our new instrument endeavored to measure identification with the five peer crowds measured in the U.S. teen I-Base Survey—Alternative, Country, Hip Hop, Mainstream, and Popular. Second, it was critical that the new instrument both measure strength of peer crowd identification as well as allow for assignment of individuals to a single peer crowd based on their strongest identification. This would allow us to reflect the multifaceted nature of teen identity and values-adoption, and also apply a range of analytical approaches to the data (9). Third, we wanted to improve upon existing instruments by creating a standardized survey less impacted by geographic and demographic differences. Fourth, the instrument needed to distinguish crowds with distinct health behavior profiles without asking directly about behavior, to avoid building a segmentation instrument around a particular health behavior rather than psychographics.

Study design was influenced by the methods we selected to examine instrument validity and predictive ability. Using a multi-trait multi-method matrix, we aimed to assess convergent validity, a metric of whether each subscale of the V-MLS measured the crowd we believed it measured, and discriminant validity, a metric of whether each crowd subscale did not measure identification with other crowds (90, 91). To complete the matrix, we examined the correlation between two methods (I-Base Survey and V-MLS) that measured the same set of traits (identification with five peer crowds). As such, participants completed the I-Base Survey during screening, and the V-MLS immediately after qualifying, providing assent/consent, and beginning the full questionnaire. Participants then completed a series of questions about health behavior, adversity, resilience, and personal traits to assess the ability of the V-MLS to predict these characteristics, which were selected based on their relevance to the creation, framing, and tone of health communication campaigns. Details on specific survey measures are provided below.

### Data collection

Data were collected in August-October 2020 from an online convenience sample of Virginia teens ages 13–18 (*N* = 1,113). Participants were recruited through paid social media advertisements (*n* = 876), outreach to previous study participants (*n* = 209), and snowball sampling (*n* = 28). Advertisements were placed on Instagram and Facebook using age and geo-targeting to minimize potential for ineligible individuals to encounter the screener. For snowball sampling, youth who completed a valid screener and questionnaire were asked to share the survey link with friends. All individuals completed an online screener survey to determine eligibility (age 13–18, Virginia resident). The screener survey also included the I-Base Survey to allow us to monitor recruitment to ensure relatively even representation of each crowd in the sample. Eligible individuals provided electronic assent/consent and were emailed a parental notification form before beginning the questionnaire. Individuals who completed a valid questionnaire received an electronic gift card incentive; the incentive amount started at $10 (*n* = 478), and increased to $15 (*n* = 509) then $20 (*n* = 126) to encourage survey completion. The study protocol and assent/consent procedures were reviewed and approved by Advarra IRB (Pro00038832).

### Measures

To enable completion of the multi-trait multi-method matrix for validity assessment, participants completed both the I-Base Survey (during screening) and the V-MLS (in the questionnaire). Participants completed the I-Base Survey by viewing a grid of teen photos and selecting three that best and three that least fit with their group of friends, then repeating the process with a second grid (11, 36). Each photo represented a single peer crowd based on prior qualitative research, and participants earned positive points for the peer crowds of photos selected as best fit and negative points for the crowds of photos selected as least fit. This resulted in a peer crowd score ranging from−12 to 12 for each of the five measured crowds (Alternative, Country, Hip Hop, Mainstream, Popular) for each participant. Additionally, participants were assigned to a single exclusive peer crowd based on their highest score. Participants completed the version of the I-Base Survey that was used in the 2019 Virginia Youth Survey (VYS), the state's implementation of the Youth Risk Behavior Survey (YRBS) (92).

**Figure 1:**
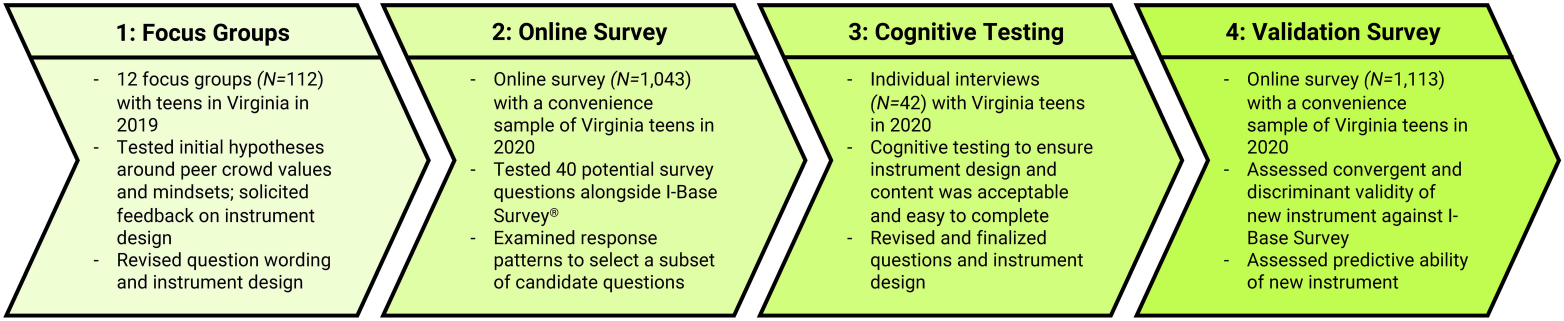
Development of the Virginia's Mindset Lens Survey.

The V-MLS was developed through three phases of formative research in Virginia (Figure 1). In Fall 2019 we conducted 12 focus groups with 112 teens to test initial hypotheses surrounding peer crowd values and mindsets. In Spring 2020 we fielded an online survey with 1,043 youth to test potential survey questions with a larger sample. Finally, in Summer 2020 we conducted 42 cognitive testing interviews to identify potential sources of measurement bias and ensure the instrument was acceptable and easy to complete (93). This process resulted in the seven-question V-MLS examined here. Each question of the V-MLS presented five response options (one hypothesized per peer crowd) and participants selected the #1 and #2 options that best described themselves or their friends. The V-MLS was completed immediately after screening and assent/consent, with question and response option order randomized within the section. We scored participants' responses by assigning 2 points for the peer crowd of responses selected as rank #1 and 1 point for the crowd of responses selected as rank #2 (with the exception of Alternative and Mainstream peer crowd responses, which earned participants 0.5 points when selected as rank #2 to counter desirability biases associated with some of the responses for these crowds). Participants received a score from 0–14 for each crowd, and we also assigned them to a single crowd based on their highest score. Details of final question wording for the V-MLS and administration support are available upon request. Questions covered core personal values and mindsets expressed by formative research participants who identified with each peer crowd: Alternative (creativity/arts, activism, individuality, challenging norms), Country (personal liberty/rights, simple living, connection to outdoors, patriotism), Hip Hop (overcoming struggles, grinding/hustling, confidence, strength), Mainstream (helping others, rules/stability, learning/personal growth, achievement), and Popular (excitement, socializing, outgoing, living life to the fullest).

To assess the V-MLS's capacity to segment peer crowds with unique health behaviors and personal traits relevant to the development of equitable health communication campaigns, participants then completed a series of behavior, experience, and trait questions. Using questions from the 2019 VYS (92), participants reported the number of days in the past 30 on which they had used cigarettes, little cigars and cigarillos (LCCs), smokeless tobacco, vapes, alcohol, and marijuana (dichotomized to yes/no), and reported if they had felt sad or hopeless for two or more weeks in the past year (yes/no). Behavior questions were selected based on having consistent associations with specific peer crowds (11, 40, 41).

Participants also completed measures of adverse childhood experiences (ACEs) as well as resilience, an individual's ability to bounce back following trauma. ACEs and resilience have been linked to a range of health outcomes (94–96), and peer crowds report differential levels of ACEs (40). We measured ACEs using an adapted version of the National Survey of Children's Health scale used previously to examine ACEs among teen peer crowds (40). Participants reported if they had experienced each of nine events, earning a score from 0–9 representing how many they had experienced: difficulty getting by on family's income, parental divorce/separation, parental incarceration, parental death, household mental illness, household substance misuse, household violence, neighborhood violence, and being treated unfairly because of race or ethnicity. Resilience was measured using the Brief Resilience Scale (BRS), a validated six-item scale that results in a score from 1–5, with a higher score indicating greater resilience (97).

Additionally, participants completed two validated personal trait scales. Participants completed the social prioritization index (SPI), a measure of the importance of socializing in an individual's life which is associated with tobacco use (83, 98). We used the eight-item abbreviated SPI, which results in a score from 0–10 with a higher score indicating greater social prioritization (98). Participants also completed the Brief Sensation Seeking Scale (BSSS) (99), an eight-item scale measuring sensation seeking, a predictor of risky behavior (100, 101). BSSS scores ranged from 1–5, with a higher score indicating greater sensation-seeking.

Finally, we collected demographic data including age in years; gender (“female”; “male”; and “transgender” and “a different identity” collapsed to “another identity” for analysis); region of Virginia (Northern, Central, Southwest, or Southeast, determined *via* zip code); and race/ethnicity (categorized as Hispanic and non-Hispanic white, Black, Asian-Pacific Islander, and other/multiracial for analysis).

### Analysis

To ensure data integrity, we conducted extensive fraud reviews during and after data collection. Age and geo-targeting of social media ads limited study exposure to those most likely to qualify, and eligibility criteria were not stated in the ads or during screening to reduce fraudulent survey attempts. Additionally, participants did not learn their eligibility status until they had completed the entire screener to reduce attempts at gaming the survey. Data quality checks identified and removed duplicate, low quality, and fraudulent cases. All analyses were conducted using SPSS Statistics Subscription for Mac (IBM Corp., Armonk, NY, United States). We first examined frequencies and means to describe the study sample.

We assessed convergent and discriminant validity by comparing scores from the V-MLS and I-Base Survey using a multi-trait multi-method matrix. This approach examines the reliability of each peer crowd score measure as well as all correlations between crowd scores measured by the same and different surveys. To complete the matrix, we first examined reliability using the OMEGA macro for SPSS to calculate McDonald's omega (ω) for each peer crowd for the I-Base Survey and V-MLS (102, 103). We then examined Pearson correlation coefficients between all I-Base Survey and V-MLS peer crowd scores. Assessments for convergent validity focused on the correlation coefficients between I-Base Survey and V-MLS scores for a single crowd. Convergent validity was indicated if these coefficients were statistically significant and positive, demonstrating convergence between the two surveys for that crowd. Assessments for discriminant validity focused on the remaining correlation coefficients in the matrix, representing the associations between scores for two different crowds. Discriminant validity was indicated if these correlation coefficients were non-significant, statistically significant and negative, or statistically significant and positive but weaker than the convergent validity coefficients for the same crowds.

We assessed predictive ability by using I-Base Survey and V-MLS scores independently as predictors of health behavior, ACEs, resilience, and personal traits *via* a series of logistic regressions (health behaviors) and linear regressions (all other characteristics). All regressions controlled for age, gender, and race/ethnicity. We compared results for the two instruments to examine the V-MLS's predictive ability compared to the established I-Base Survey for these characteristics that are key to health communication campaign tailoring. This allowed us to understand if the V-MLS successfully segmented teens into distinct crowds with unique profiles that could be effectively reached with a uniquely tailored and targeted campaign.

## Results

Sample descriptives are presented in Table 2. The racial/ethnic distribution of the sample mirrored census data for Virginia, and just over half identified as female. The proportion of the sample scoring highest for each peer crowd was largely similar between the I-Base Survey and V-MLS. Health behavior responses mirrored those reported in the 2019 VYS (104).

**Table 2:** Participant characteristics.

**Characteristic**	***n*** **or** ***M***	**% or (SD)**	**Characteristic**	***n*** **or** ***M***	**% or (SD)**
**Gender**	***n***	**%**	**Region of Virginia**	***n***	**%**
Female	638	57.3	North	357	32.1
Male	393	35.3	Central	301	27.0
Another identity	82	7.4	Southeast	236	21.2
**Race/ethnicity**	***n***	**%**	**Southwest**	**219**	**19.7**
Hispanic	125	11.2	Behaviors/risks	*n*	%
NH white	606	54.4	Current cigarette use	87	7.8
NH Black	180	16.2	Current LCC use	54	4.9
NH API	92	8.3	Current smokeless tobacco use	27	2.4
NH other/multiracial	110	9.9	Current vape use	207	18.6
Age	*n*	%	Current alcohol use	323	29.0
13–15	214	19.2	Current marijuana use	223	20.0
16–18	899	80.8	Felt sad or hopeless	639	57.4
**I-Base Survey peer crowd**	***n***	**%**	**V-MLS peer crowd**	***n***	**%**
Alternative	291	26.1	Alternative	338	30.4
Country	152	13.7	Country	118	10.6
Hip Hop	144	12.9	Hip Hop	162	14.6
Mainstream	191	17.2	Mainstream	207	18.6
Popular	215	19.3	Popular	210	18.9
Tied	120	10.8	Tied	78	7.0
**Experiences**	***M***	**(SD)**	**Personal traits**	***M***	**(SD)**
ACE score (0–9)	2.85	(2.14)	SPI score (0–10)	3.39	(2.22)
Resilience score (1–5)	3.01	(0.81)	Sensation-seeking score (1–5)	3.33	(0.76)

*ACE, adverse childhood experience; API, Asian-Pacific Islander; LCCs, little cigars and cigarillos; NH, non-Hispanic; SPI, social prioritization index; V-MLS, Virginia's Mindset Lens Survey*.

We assessed convergent and discriminant validity using a multi-trait multi-method matrix (Table 3). Reliability, shown in the matrix diagonal, was consistently higher for the V-MLS than the I-Base Survey, with McDonald's ω values for the new survey (0.56–0.77) falling in or near the acceptable range for scales with fewer than 10 items (0.60 or higher) (105). Correlations between I-Base Survey and V-MLS scores for a given peer crowd were statistically significant and positive for all five crowds (convergent validity), ranging from 0.17 (Hip Hop) to 0.55 (Country). With one exception, all other correlations in the matrix (discriminant validity) were negative, not statistically significant, or positive but weaker than the corresponding convergent validity correlations. The correlation coefficient between I-Base Survey Hip Hop score and V-MLS Popular score was 0.18 (*p* < 0.001), which was weaker than the coefficient between the two Popular scores (0.26, *p* < 0.001) but marginally stronger than the coefficient between the two Hip Hop scores (0.17, *p* < 0.001). Overall, the V-MLS demonstrated convergent and discriminant validity.

**Table 3:** Multi-trait multi-method matrix for I-Base Survey and V-MLS.

		**I-Base Survey**					**V-MLS**				
		**A**_1_	**C**_1_	**H**_1_	**M**_1_	**P**_1_	**A**_2_	**C**_2_	**H**_2_	**M**_2_	**P**_2_
**I-Base Survey**	**A_**1**_**	ω = 0.78	**-**	**-**	**-**	**-**	**-**	**-**	**-**	**-**	**-**
	**C_**1**_**	−0.60***	ω = 0.77	**-**	**-**	**-**	**-**	**-**	**-**	**-**	**-**
	**H_**1**_**	−0.23***	−0.25***	ω = 0.48	**-**	**-**	**-**	**-**	**-**	**-**	**-**
	**M_**1**_**	−0.23***	−0.14***	−0.27***	ω = 0.44	**-**	**-**	**-**	**-**	**-**	**-**
	**P_**1**_**	−0.59***	−0.01	0.09**	0.05	ω = 0.45	**-**	**-**	**-**	**-**	**-**
**V-MLS**	**A_**2**_**	0.53***	−0.40***	−0.06*	−0.03	−0.32***	ω = 0.70	**-**	**-**	**-**	**-**
	**C_**2**_**	−0.30***	0.55***	−0.12***	−0.14***	**−0.03**	−0.38***	ω = 0.77	**-**	**-**	**-**
	**H_**2**_**	−0.17***	0.08**	0.17***	−0.08*	0.11***	−0.36***	−0.05	ω = 0.56	**-**	**–**
	**M_**2**_**	−0.02	−0.07*	−0.13***	0.23***	0.06*	−0.22***	−0.26***	−0.23***	ω = 0.61	**-**
	**P_**2**_**	−0.19***	−0.04	0.18***	−0.02	0.26***	−0.28***	−0.19***	−0.17***	−0.28***	ω = 0.59

*A, Alternative; C, Country; H, Hip Hop; M, Mainstream; P, Popular; V-MLS, Virginia's Mindset Lens Survey*.

*Boldface indicates statistical significance (p < 0.05)*.

*p < 0.05 **p < 0.01 ***p < 0.001.

*The shaded values used to visually distinguish the diagonal (which contains omega values) from the other cells (which contain correlation coefficients)*.

To examine predictive ability, we conducted a series of regressions using I-Base Survey and V-MLS peer crowd scores to separately predict behavior, ACEs, resilience, and personal traits while controlling for demographics (Table 4). Results for the two surveys largely aligned. Higher Hip Hop scores on both surveys were associated with significantly increased odds of current LCC, vape, and marijuana use, and higher ACE scores. For Alternative, higher scores on both surveys were associated with significantly increased odds of current marijuana use and feeling sad or hopeless; reduced odds of current smokeless tobacco use; higher ACE and sensation-seeking scores; and lower resilience scores. Higher Popular scores on both surveys were associated with increased odds of current alcohol use, higher SPI scores, and lower ACE scores. For Country, higher scores on both surveys were associated with significantly increased odds of current cigarette and smokeless tobacco use, reduced odds of feeling sad or hopeless, and lower sensation-seeking scores. Higher Mainstream scores on both surveys were associated with significantly reduced odds for all behaviors except feeling sad, and with lower ACE, SPI, and sensation-seeking scores. Similar patterns across surveys demonstrated that the V-MLS has sufficient capability to predict key characteristics associated with peer crowds that should inform targeted health communication campaign development.

**Table 4:** Peer crowd scores as predictors of behavior, experiences, and personal traits.

**Score**	**Alternative**		**Country**		**Hip Hop**		**Mainstream**		**Popular**	
**Instrument**	**I-Base Survey**	**V-MLS**	**I-Base Survey**	**V-MLS**	**I-Base Survey**	**V-MLS**	**I-Base Survey**	**V-MLS**	**I-Base Survey**	**V-MLS**
**Behavior (AOR)**										
Cigarettes	1.02	0.98	1.05*	1.10**	1.04	1.04	0.79***	0.79***	0.94*	1.07
LCCs	1.01	0.93	1.04	1.12**	1.16***	1.13*	0.75***	0.77***	0.94	1.05
Smokeless tobacco	0.94*	0.81**	1.22***	1.29***	1.04	1.14	0.70***	0.79*	0.92	0.84*
Vapes	1.00	0.98	1.02	1.05	1.09***	1.09**	0.84***	0.81***	1.00	1.09***
Alcohol	0.99	0.99	1.00	1.01	1.06**	1.04	0.92***	0.89***	1.04*	1.08***
Marijuana	1.04***	1.05*	0.96*	0.99	1.08***	1.06*	0.86***	0.80***	0.96	1.08**
Felt sad/ hopeless	1.08***	1.17***	0.94***	0.91***	1.01	0.92***	0.92***	1.01	0.93***	0.96
**ACE score (B)**	0.06***	0.09***	−0.04***	−0.04	0.05**	0.07**	−0.12***	−0.08***	−0.08***	−0.05*
**Resilience score (B)**	−0.02***	−0.03***	0.00	0.02*	0.01	0.03***	0.02*	−0.02	0.03***	0.01
**SPI score (B)**	−0.02*	−0.02	0.01	0.05*	0.09***	−0.02	−0.11***	−0.29***	0.04*	0.30***
**Sensation-seeking score (B)**	0.01*	0.02**	−0.01**	−0.02*	0.03***	0.00	−0.04***	−0.08***	0.00	0.08***

*ACE, adverse childhood experience; AOR, adjusted odds ratio; LCCs, little cigars and cigarillos; SPI, social prioritization index; V-MLS, Virginia's Mindset Lens Survey*.

*For linear regressions, table presents unstandardized coefficients (B). All regressions control for age, gender, and race/ethnicity*.

*Boldface indicates statistical significance (p < 0.05)*.

*p < 0.05 **p < 0.01 ***p < 0.001.

*The shaded values used to visually identify which pairs of columns go together (i.e., to keep the I-Base Survey and V-MLS columns for a particular peer crowd together visually for easy comparison)*.

## Discussion

The current manuscript describes validation and predictive ability testing for the new Mindset Lens Survey in Virginia, a mindset-based teen peer crowd segmentation survey that groups teens into crowds with distinct behavioral and psychographic characteristics. The V-MLS represents an evolution in peer crowd measurement for health communications segmentation as it more directly segments youth based on the values and mindsets that may influence peer crowd behavior. By using psychographics to segment teens into groups with real-world social influence, the V-MLS provides health marketers with the audience insights necessary to create targeted, tailored health campaigns that can more equitably address disparities.

The V-MLS demonstrated reliability and convergent and discriminant validity, elements that to our knowledge have not been reported for existing peer crowd measurement tools. Importantly, peer crowd risk profiles from the V-MLS align with existing literature, further reinforcing the validity of the new tool. Specifically, associations previously reported between the Alternative peer crowd and marijuana use, mental health struggles, and ACEs; the Country peer crowd and smokeless tobacco; the Hip Hop peer crowd and tobacco use, substance use, and ACEs; the Mainstream peer crowd and reduced risk across behaviors; and the Popular peer crowd and vaping, alcohol, and reduced ACEs were also observed when using the V-MLS (11, 28, 34, 35, 40, 41, 45, 47).

As with any new instrument, while the V-MLS demonstrates strong reliability and validity, it does not exactly match results derived from existing methods. In the regressions assessing predictive ability, there were several instances in which one of the survey tools produced a statistically significant result while the other did not. In most of these cases, however, the non-significant result trended in the same direction as the significant result, indicating the disparity may be due to power limitations. Generally, results of regression analyses indicate that the V-MLS is effective at identifying crowds with distinct behaviors, experiences, and traits, information that can inform health communication campaign development. In fact, results may indicate that we created a survey that more directly taps into the core psychographic characteristics that differentiate peer crowds, compared to indirect methods. Upcoming research efforts applying the instrument to representative teen surveys in Virginia and other states will allow us to further explore this issue and to demonstrate its generalizability to U.S. states beyond Virginia.

Based on the strength of our findings, we believe the V-MLS represents progress in peer crowd measurement methods and will be a useful tool for health communication campaign segmentation. The instrument is brief, acceptable to respondents, and can easily be appended to ongoing data collections such as representative surveys to inform audience segmentation. It allows youth to pick a primary and secondary response for each question, adding depth to our understanding by providing insight into the intensity of agreement with the values and the peer crowds they represent. Additionally, the V-MLS brings researchers one step closer to directly measuring the core characteristics that differentiate peer crowds—values, mindsets, and worldviews—making it a more efficient means of gathering insightful peer crowd data. The V-MLS provides information critical to effective health communication by revealing the values and mindsets that characterize each peer crowd and that should be incorporated into message framing for a peer crowd-targeted campaign (9, 14, 83). Per the mindsponge model, the values that pass through a teen's filters and comfort zone to reach their core beliefs will influence their behavior and reinforce acceptance of similar values and mindsets in the future (23, 24). By framing health messages based on the values and interests shared by teens within higher-risk audience segments, campaigns can increase the effectiveness of their messages through greater tuning in from the right audience, stronger message receptivity once processed, and ultimately a greater impact on behavior (9). These improvements will increase a campaign's ability to persuade those at higher risk, making the campaign's outcomes more equitable.

While critical to successful message development, information provided by the V-MLS does not address other important elements of campaign development such as the look, style, and feel of the campaign or the media channels where content should be placed (9, 14). As such, we believe that peer crowd visual cue data such as that provided by the I-Base Survey or qualitative data collection remains important to inform the style of campaign ads and actors, while data on media channel preferences should also be collected to ensure tailored content efficiently reaches the target crowd (83). Together, these data can provide the detailed portrait of teen peer crowds necessary to inform targeted health communication campaigns. Additionally, health communication campaigns should still rely on information processing frameworks such as 3D creativity management theory or prototype-willingness model, and campaign design frameworks such as Social Branding or social marketing benchmark criteria to translate audience insights into effective campaigns (14, 63, 106–108).

### Limitations

We have several limitations to note. Because data come from a convenience sample of Virginia teens, results may not generalize beyond the current sample or state. However, given consistent similarities in peer crowds observed across states (9–11, 37, 41), we anticipate the instrument will perform effectively in other U.S. states. Cross-cultural adaptation to reflect local crowds and crowd values would be necessary to ensure applicability to other countries, where crowds and their interests may differ (29, 31, 32, 39, 44–47, 49). Some McDonald's ω values for the V-MLS were lower than typically expected for validated instruments, though still acceptable given the small number of survey items included in the final instrument and the instrument's design (105). In order to complete our validation analyses, we had to compare the V-MLS to an existing peer crowd measurement tool and selected the I-Base Survey for this task. Though the I-Base Survey does not have published validation results, we felt this was the best available comparison point as no “gold standard” instrument exists and the I-Base Survey has been used extensively with consistent findings, demonstrating applied validity (11, 13, 36, 37, 41, 82). As our data were cross-sectional we could not track changes in values over time, but future work could seek to extend the application of the mindsponge model to peer crowds by exploring how peer crowd values enter and leave a teen's core beliefs and the impact this has on behavior.

We observed a significant, positive association between I-Base Survey Hip Hop score and V-MLS Popular score, which was marginally stronger than the association between the two Hip Hop scores. Additionally, the correlation between the two Hip Hop scores was relatively lower than other intra-crowd correlations. We believe this reflects the increasing overlap between the Hip Hop and Popular crowds—particularly in terms of personal style as captured *via* the I-Base Survey—which has emerged due to the rapid increase in popularity of hip hop music and culture in the past decade (109). As the Popular crowd are trendsetters who adopt the latest fashion and music styles, the rise of hip hop culture's popularity has blurred stylistic differences between the crowds, but may not have impacted crowd values and mindsets. We feel this concern is outweighed by the strong predictive power of V-MLS scores for Hip Hop and Popular. Combined with the instrument's focus on values and mindsets that directly impact behavior, findings may indicate the V-MLS more effectively differentiates these two related crowds than prior instruments.

We also must acknowledge that our approach was more confirmatory than exploratory. We did not start with a blank slate and rely on a methodology such as cluster analysis to create new crowds. Instead, we started with a concept of five teen peer crowds based on the literature. We believe this approach was warranted as we aimed to improve upon available methods rather than reinvent decades of convergent research. Additionally, during early qualitative data collection we included participants who did not fall neatly into a single existing peer crowd to allow for the potential emergence of new crowds. However, evidence did not support adding a new crowd to those previously observed. Our chosen approach ensured that the V-MLS identifies groups of youth who not only share characteristics but also influence each others' behavior through social norms, a critical feature if the instrument is to be useful in health communications segmentation. A cluster analysis would not have guaranteed the creation of crowds with meaningful shared norms and culture, which led us to adopt the approach described herein.

## Conclusions

This manuscript describes the validity and predictive ability of Virginia's Mindset Lens Survey, a new mindset-based teen peer crowd segmentation survey that complements existing methods to provide audience insights necessary to create peer crowd-targeted health communication campaigns. The V-MLS addresses many of the shortcomings of previous peer crowd measurement tools when applied to audience segmentation, and demonstrates reliability and validity. The survey is a brief and practical instrument that can be appended to surveillance surveys and, when combined with relevant information processing and campaign design frameworks, can inform the design and tailoring of health campaigns for higher-risk teens alongside complementary instruments providing visual cue and media use data. Future research seeks to replicate and extend findings in representative samples in Virginia and other U.S. states, explore cross-cultural adaptation opportunities, and apply the survey to real-world health communication campaign development.

## Data availability statement

The raw data supporting the conclusions of this article will be made available by the authors, without undue reservation.

## Ethics statement

The studies involving human participants were reviewed and approved by Advarra IRB. Written informed consent from the participants' legal guardian/next of kin was not required to participate in this study in accordance with the national legislation and the institutional requirements.

## Author contributions

All authors conceived of and designed the research study. CS and MD implemented the research. CS analyzed the data and wrote the first draft. All authors contributed to article revisions and approved the submitted version.

## Funding

This research was funded by the Virginia Foundation for Healthy Youth.

## Conflict of interest

Authors CS, JJ, and MD are employed by Rescue Agency, a research and campaign contractor for the Virginia Foundation for Healthy Youth, the study sponsor and employer of authors DS and HB. Rescue Agency holds the proprietorship and trademark for the I-Base Survey and Mindset Lens Survey used in this manuscript. As employees of the funding agency, the Virginia Foundation for Healthy Youth, DS and HB were involved in study design and manuscript writing.

## Publisher's note

All claims expressed in this article are solely those of the authors and do not necessarily represent those of their affiliated organizations, or those of the publisher, the editors and the reviewers. Any product that may be evaluated in this article, or claim that may be made by its manufacturer, is not guaranteed or endorsed by the publisher.

## Abbreviations

ACE: adverse childhood experiences
AOR: adjusted odds ratio
API: Asian-Pacific Islander
BRS: Brief Resilience Scale
BSSS: Brief Sensation Seeking Scale
LCC: little cigars and cigarillos
NH: non-Hispanic
SPI: Social Prioritization Index
V-MLS: Virginia's Mindset Lens Survey
VYS: Virginia Youth Survey
YRBS: Youth Risk Behavior Survey.
