# Supplementary material for: Psychographic segmentation to identify higher-risk teen peer crowds for health communications: Validation of Virginia's Mindset Lens Survey
Source: Front Public Health. 2022 Jul 22;10:871864. doi: 10.3389/fpubh.2022.871864 (PMC9355138; doi:10.3389/fpubh.2022.871864)
Supplement: Supplementary file 1 [file Data_Sheet_1.pdf]

Figure S1. Peer Crowd-Targeted Campaign Examples

Peer Crowd	Campaign	Example Content	
Hip Hop	*Hustle & Strive* (substance use; Virginia)	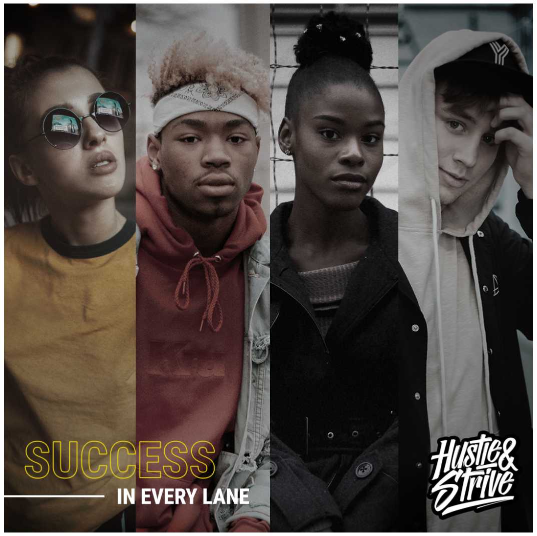	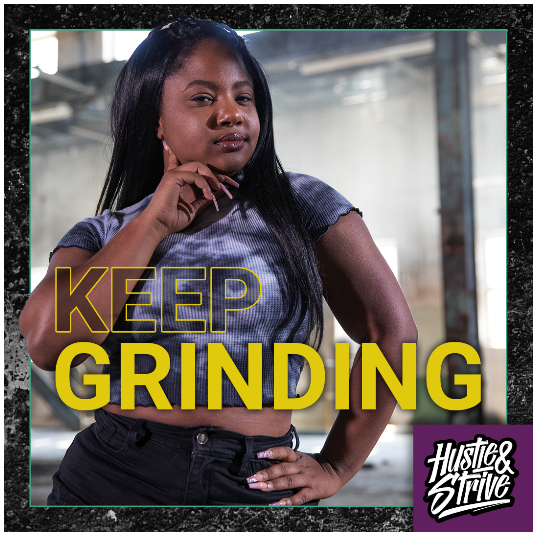
Alternative	*Syke* (tobacco use; Virginia)	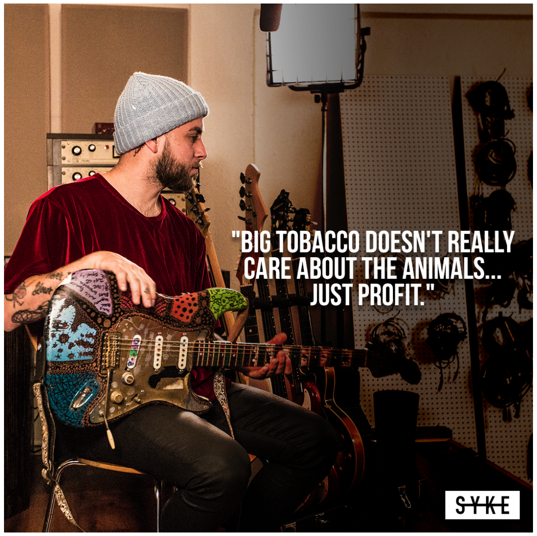	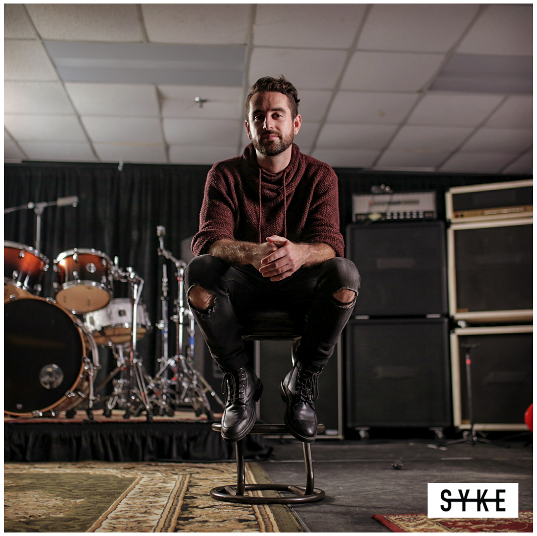

<table>
<tr><th>Peer Crowd</th><th>Campaign</th><th>Example Content</th><th></th></tr>
<tr><td>Country</td><td>Down & Dirty
(tobacco use; multiple states)</td><td>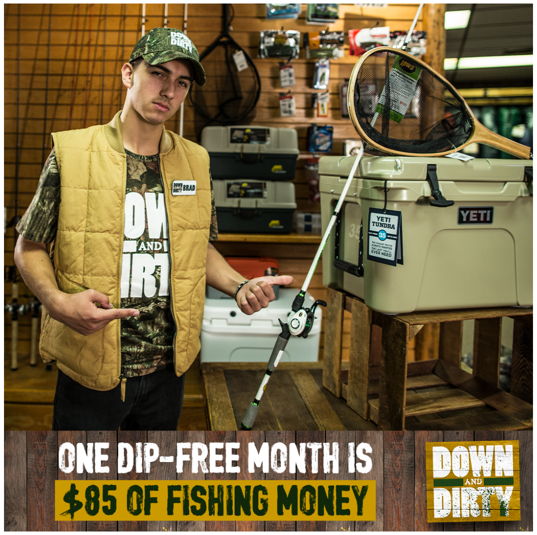
</td><td>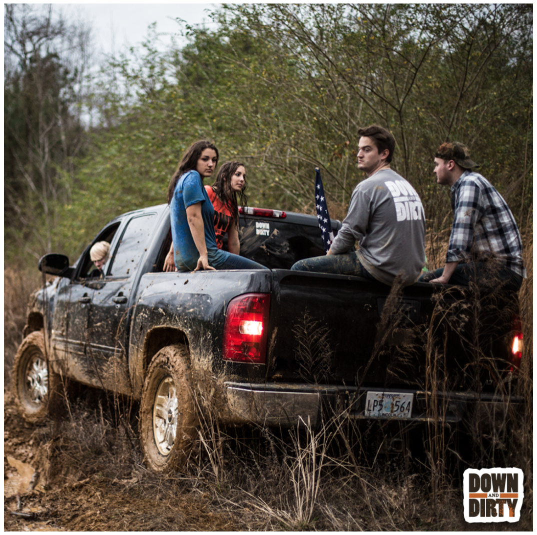
</td></tr>
<tr><td>Mainstream</td><td>Y Street, Evolvement
(youth engagement programs; multiple states)</td><td>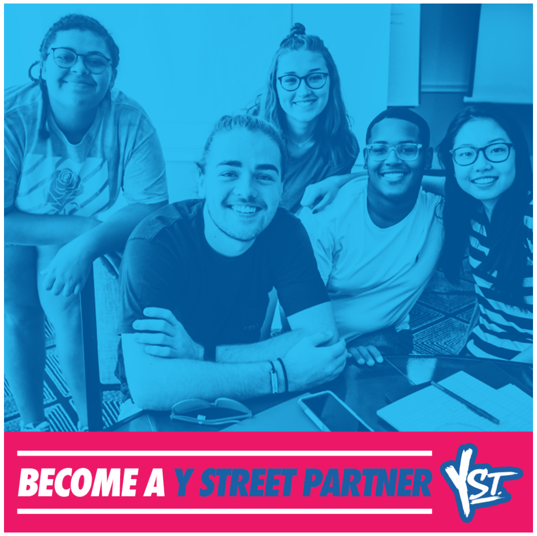
</td><td>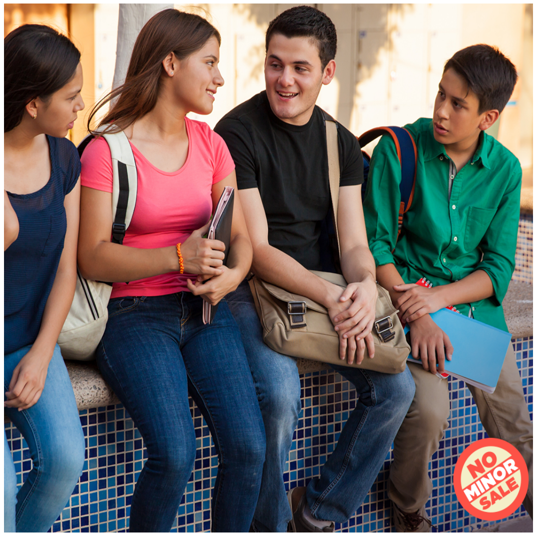
</td></tr>
</table>

Peer Crowd	Campaign	Example Content
Popular	*Behind The Haze* (vaping; multiple states)	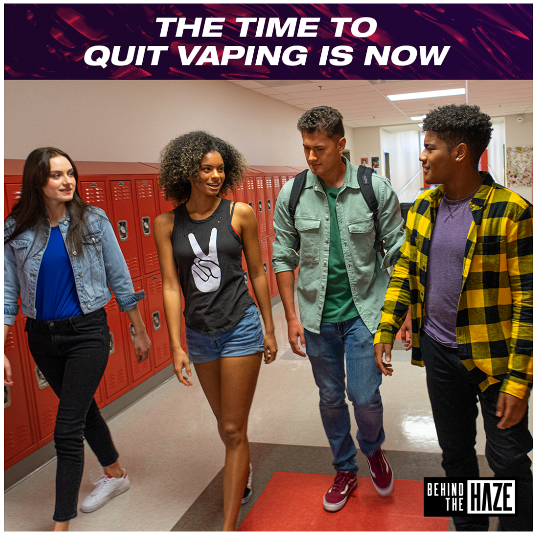 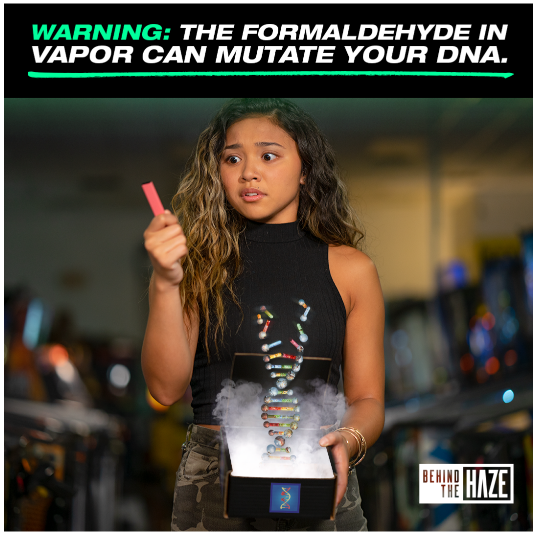
